# Combined regional T1w/T2w ratio and voxel-based morphometry in multiple system atrophy: A follow-up study

**DOI:** 10.3389/fneur.2022.1017311

**Published:** 2022-10-19

**Authors:** Sara Ponticorvo, Renzo Manara, Maria Claudia Russillo, Valentina Andreozzi, Lorenzo Forino, Roberto Erro, Marina Picillo, Marianna Amboni, Sofia Cuoco, Gianfranco Di Salle, Francesco Di Salle, Paolo Barone, Fabrizio Esposito, Maria Teresa Pellecchia

**Affiliations:** ^1^Neuroscience Section, Department of Medicine, Surgery and Dentistry, Scuola Medica Salernitana, University of Salerno, Salerno, Italy; ^2^Neuroradiology Unit, Department of Neurosciences, University of Padua, Padua, Italy; ^3^Institute of Life Sciences, Scuola Superiore Sant'Anna, Pisa, Italy; ^4^Department of Advanced Medical and Surgical Sciences, University of Campania “Luigi Vanvitelli”, Naples, Italy

**Keywords:** multiple system atrophy, disease progression, T1w/T2w ratio, MRI markers, neurodegeneration

## Abstract

Several MRI techniques have become available to support the early diagnosis of multiple system atrophy (MSA), but few longitudinal studies on both MSA variants have been performed, and there are no established MRI markers of disease progression. We aimed to characterize longitudinal brain changes in 26 patients with MSA (14 MSA-P and 12 MSA-C) over a 1-year follow-up period in terms of local tissue density and T1w/T2w ratio in a-priori regions, namely, bilateral putamen, cerebellar gray matter (GM), white matter (WM), and substantia nigra (SN). A significant GM density decrease was found in cerebellum and left putamen in the entire group (10.7 and 33.1% variation, respectively) and both MSA subtypes (MSA-C: 15.4 and 33.0% variation; MSA-P: 7.7 and 33.2%) and in right putamen in the entire group (19.8% variation) and patients with MSA-C (20.9% variation). A WM density decrease was found in the entire group (9.3% variation) and both subtypes in cerebellum-brainstem (MSA-C: 18.0% variation; MSA-P: 5% variation). The T1w/T2w ratio increase was found in the cerebellar and left putamen GM (6.6 and 24.9% variation), while a significant T1w/T2w ratio decrease was detected in SN in the entire MSA group (31% variation). We found a more progressive atrophy of the cerebellum in MSA-C with a similar progression of putaminal atrophy in the two variants. T1w/T2w ratio can be further studied as a potential marker of disease progression, possibly reflecting decreased neuronal density or iron accumulation.

## Introduction

Multiple system atrophy (MSA) is an adult-onset progressive neurodegenerative disorder characterized by a combination of autonomic failure, parkinsonism, and cerebellar ataxia (1). It can be classified into the parkinsonian variant (MSA-P) and the cerebellar variant (MSA-C) based on clinical presentation, which is dependent on the distribution of pathology within the basal ganglia and the olivopontocerebellar system (1). MSA is a rapidly progressive disease (2–4); indeed, the predominant motor features can change with time and the variability of the severity and regional distribution of pathological processes accounts for a spectrum of diseases. Several studies have focused on supporting the early diagnosis of MSA, but only few longitudinal studies have dug into the analysis of progression to eventually find markers of disease course. The progression of structural brain damage (e.g., cortical and subcortical atrophy) can be studied *in-vivo* using magnetic resonance imaging (MRI) methods in order to evaluate the relationships with clinical features and disease course (5–7). Previous findings have already estimated annual rates of atrophy and signal alterations in specific regions, only partially correlating with clinical progression (4, 8). Furthermore, clinical MSA subtypes (MSA-C and MSA-P) can differently evolve over time in terms of type or spatial localization of detectable brain damage. In the last decades, not routinary MRI acquisition methods and processing have been developed to obtain quantitative biomarkers of brain tissue composition or macromolecular content [refer to, e.g., the recent review of Weiskopf et al. (9)]; these methods provide specific physical parameters, which carry information to directly characterize biological tissue structure, but they are not always easily applicable to study clinical populations due to long acquisition times and specific MRI scanner technical requirements. Therefore, also other methods that rely on clinical routinely MRI acquisitions have been proposed. One of the simplest methods is to calculate the ratio of the signal intensity of a T1-weighted and a T2-weighted (T1w/T2w) MRI image, which has been proposed as a proxy of cortical myelin content (10–12) but that is also influenced by other factors such as iron content (11, 13, 14), axon, and dendrite density. Consistently, an increased T1w/T2w ratio has been recently found in the substantia nigra (SN) pars compacta of patients with Parkinson's disease (PD) and MSA compared with healthy controls (HCs) (15, 16).

As compared with quantitative acquisition methods, the T1w/T2w ratio has the advantage that can be easily obtained, since T1w and T2w are routinely acquired MRI sequences in standard clinical sessions.

Starting from our previous cross-sectional study that assessed brain atrophy and brain tissue integrity by means of voxel-based morphometry (VBM) and T1w/T2w ratio in patients with MSA (16), in this study, we aimed to characterize the longitudinal changes in the same regions of interest (ROIs) in the same group of patients with MSA over time in order to possibly establish a marker for disease progression and shed light on differentiation of brain alterations course in the two clinical variants. In particular, we evaluated disease progression in both gray matter (GM) and white matter (WM), as well as the T1w/T2w parameter by performing an explorative whole brain voxel-wise and an ROI-based longitudinal study in patients with both subtypes of MSA, evaluated at baseline and after 1-year follow-up. Moreover, we aimed to investigate the relationships between changes in clinical manifestations of disease severity and brain changes as detected by MRI to help evaluate the possible value of such MRI parameters as biomarkers of disease progression in MSA.

## Methods

### Subjects

A total of 26 patients with probable MSA according to current diagnostic criteria [second consensus criteria (1)], 14 with the parkinsonian variant, MSA-P, and 12 with the cerebellar variant, MSA-C, participated in this study. Disease severity was assessed with the Unified MSA Rating Scale (UMSARS). Demographic, clinical, and imaging data collection and examination were performed for all participants twice with a mean (±standard deviation) follow-up time of 375.5 days (±118.5). This study was approved by the local ethics committee (Comitato Etico Campania Sud), and all participants signed informed consent. This study was conducted in accordance with the Declaration of Helsinki principles.

### MRI acquisition

All brain imaging data were acquired on the same 3T MRI scanner (MAGNETOM Skyra, Siemens, Erlangen Germany) operated with a 20-channel head and neck coil. The imaging protocol consisted of a 3D anatomical T1-weighted (T1w) Magnetization Prepared RApid Gradient Echo (MPRAGE) sequence with repetition time (TR) = 2,400 ms and echo time (TE) = 2.25 ms, spatial resolution = 1 × 1 × 1 mm^3^, matrix size = 256 × 256, anterior-posterior phase encoding direction, generalized autocalibrating partially parallel acquisitions (GRAPPA) factor of 2 in phase-encoding direction, and a 3D T2-weighted (T2w) Sampling Perfection with Application optimized Contrast using different angle Evolutions (SPACE) sequence with TR = 3,200, TE = 408 ms, variable flip angle, resolution = 1 × 1 × 1 mm^3^, matrix size = 256 × 256, anterior-posterior phase encoding direction, and GRAPPA factor of 2 in phase-encoding.

### MRI data processing

For longitudinal analysis, the two anatomical 3D-T1w volumes of each subject (t0 and t1) were combined into one 3D-T1w average volume (T1w-avg) using the intra-subject longitudinal diffeomorphic transformation as implemented in the statistical parametric mapping toolbox (SPM12) (17). Then, all T1w-avg images were normalized to the Montreal Neurological Institute (MNI) standard space using Diffeomorphic Anatomical Registration Through Exponentiated Lie (DARTEL) algebra algorithm (18). Native T1w images were aligned to the respective T1w-avg volume with the nonlinear warping calculated in the intra-subject alignment, segmented into GM and WM maps, and resampled in the standard MNI space using the deformation fields calculated above. The resulting tissue (GM/WM) probabilistic maps were modulated by the Jacobian determinants of the deformations to account for local compression and expansion due to linear and nonlinear transformation (19) and then smoothed with a Gaussian kernel of 6 mm FWHM.

To obtain maps proxy of myelin content (T1w/T2w), before the validated preprocessing (12), T1w and T2w images were corrected for intensity nonuniformity with the bias correction tool implemented in the unified segmentation (20) and available in SPM12. Then, the T2w images were linearly registered to the T1w images using the FSL tool FLIRT (21, 22) for estimating and applying a rigid-body affine transformation with 6 degree of freedom and cubic spline interpolation to minimize the WM and CSF contamination of GM voxels (12). T1w/T2w maps were obtained using FSLMATHS to divide the T1w volumes by the corresponding aligned T2w ones. For spatial normalization, T1w/T2w maps were firstly aligned to the corresponding T1w-avg and then transformed (with the same group template and deformation fields) to the MNI space. During the normalization procedure, T1w/T2w maps were smoothed with a Gaussian kernel of 6 mm FWHM (refer to Supplementary Figure S1 for a representation of the processing workflow).

### Statistical analysis

A whole brain explorative voxel-wise analysis was performed for all maps (WM and GM density and T1w/T2w) with a paired *t*-test as implemented in SPM12 and including age and sex as covariates both considering the whole group and separating the subtypes (MSA-C and MSA-P). Statistical *t*-maps from the comparisons were thresholded by applying the family-wise error (FWE) correction for multiple comparisons using Gaussian random field theory.

Longitudinal statistical comparisons were performed on tissue density (GM and WM) and on T1w/T2w mean regional values according to previous results (16) of local atrophy in GM (left and right putamen and cerebellar GM) and in WM (cerebellum-brainstem WM) when patients with MSA were compared with a group of age-matched HC. Furthermore, an atlas-derived ROI of the bilateral SN pars compacta (freely available at https://github.com/apoorvasafai/NMS-SNc-atlas) was also included in the analysis. Thus, regional mean values of WM, GM, and T1w/T2w were extracted at the two time points (t0 and t1) for each subject, corrected with a linear regression model for age and sex, and compared using a nonparametric Wilcoxon signed-rank test both considering the entire MSA group and separating for subtypes (MSA-C and MSA-P). The percent variation of each parameter over the follow-up time was also calculated as 100^*^(value *t*1 – value *t*0)/value t0. Results were considered significant at *p* < 0.05 after Bonferroni correction for multiple comparisons. Correlations between variation (delta value (Δ) = value *t*1 – value *t*0) of the regional MRI parameters (GM density, WM density, and T1w/T2w) and variation of clinical variables (UMSARS I, UMSARS II, and UMSARS III) were also calculated using the Spearman's rank correlation coefficient. Finally, a general linear model was performed in order to assess how the value of the different MRI parameters (GM, WM density, and T1w/T2w) at baseline influences the clinical changes (in terms of UMSARS score variation), considering the disease duration as additional confound predictor.

## Results

Demographic and clinical data of enrolled subjects are reported in Table 1.

**Table 1:** Demographic and clinical findings of enrolled patients at baseline.

	**MSA**	**MSA-P**	**MSA-C**	***p***
	**(*n* = 26)**	**(*n* = 14)**	**(*n* = 12)**	
Age, ys (mean ± SD)	59.5 ± 6.7	60.9 ± 7.5	57.9 ± 5.3	*p* > 0.05
Disease duration, ys (mean ± SD)	3.6 ± 1.3	3.6 ± 1.28	3.6 ± 1.31	*p* > 0.05
UMSARS-I (mean ± SD)	22.07 ± 6	20 ± 6.1	18.3 ± 2.4	*p* > 0.05
UMSARS-II (mean ± SD)	24.07 ± 6.9	23.6 ± 7	19.8 ± 2.8	*p* > 0.05
UMSARS-IV (mean ± SD)	2.7 ± 0.8	2.6 ± 0.8	2.3 ± 0.5	*p* > 0.05

Reported p-values (last column) are referring to a statistical comparison between patient subgroups (MSA-C vs. MSA-P).

Whole brain voxel-wise analysis did not show longitudinal differences in any of the maps considered at the statistical threshold of *p* < 0.05 FWE corrected.

Previous clusters of GM/WM atrophy compared with an independent group of age-matched HCs (bilateral putamina, cerebellar GM, and cerebellum-brainstem WM) and an atlas-derived ROI of the SN were longitudinally compared in the patient cohort both considering the MSA cohort as an entire and separating MSA-C and MSA-P.

**Figure 1:**
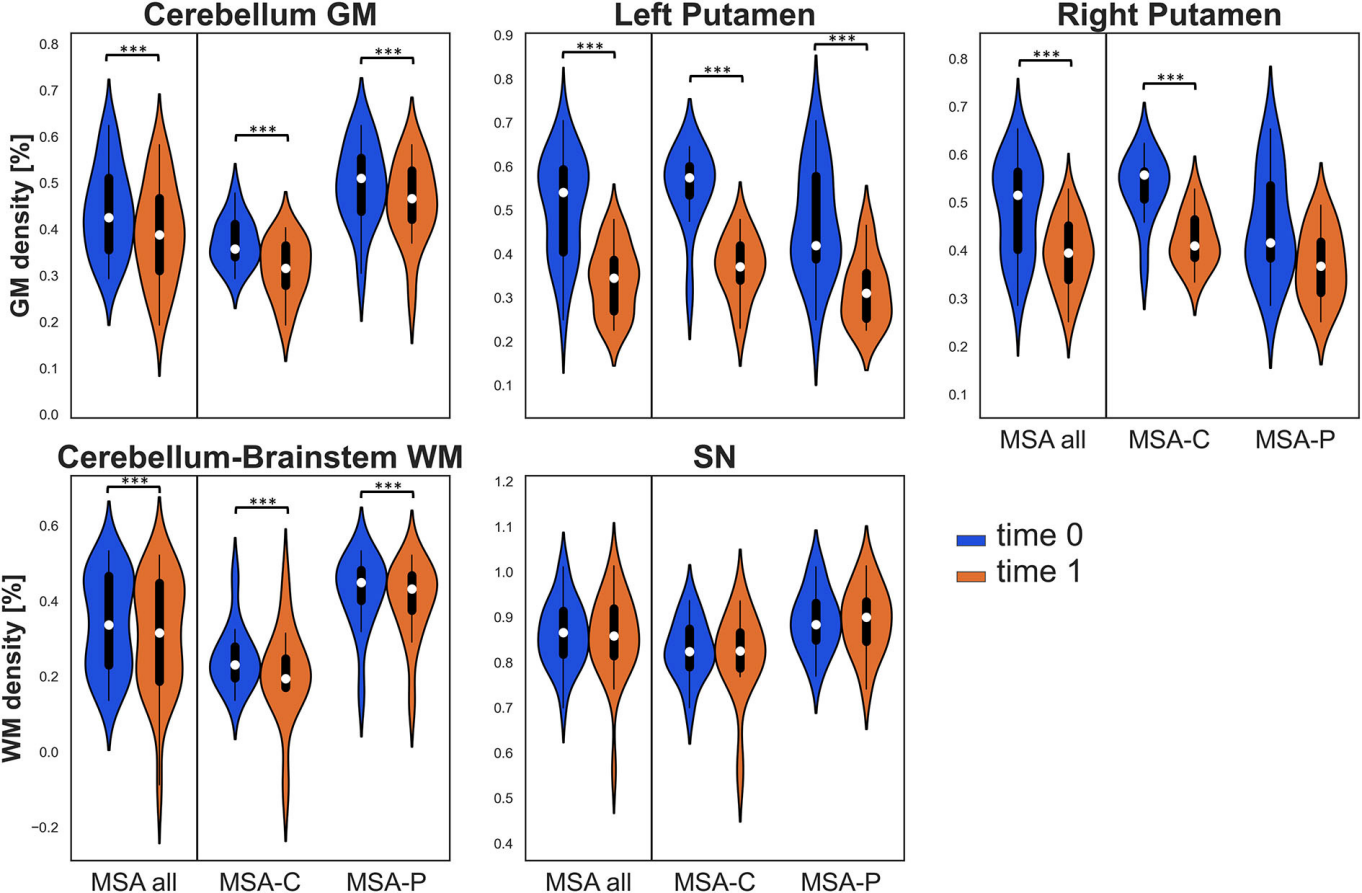
Violin plots describing distributions of regional tissue density in all the considered regions of interest (ROIs). The panels of the first row illustrate gray matter (GM) density in the clusters of the cerebellum and bilateral putamina, while the panels of the second row illustrate white matter (WM) density in the cerebellum-brainstem cluster and in the bilateral substantia nigra (SN). Values at baseline (t0) are in blue, while values at follow-up (t1) are in orange for the entire multiple system atrophy (MSA) group (MSA all) and for both MSA subgroups. Significant differences are indicated with asterisks. ***p < 0.001.

As for GM density, a significant decrease was found at follow-up in the entire group and in both MSA-P and MSA-C in the cerebellum cluster, the left putamen cluster, and in the entire group and MSA-C group also in the right putamen. As for WM density, a significant longitudinal decrease was found in all groups in the cerebellum-brainstem cluster (refer to Table 2 and Figure 1). The percent variability of atrophy (over the follow-up time) in putamina was similar in MSA-P and MSA-C, while it was higher in cerebellar GM in MSA-C as compared with MSA-P (*p* = 0.05).

**Table 2:** Results of regional longitudinal analysis.

	**MSA entire group**			**MSA-C**			**MSA-P**		
	**Uncorrected *p*-value**	**% variation**	***n***	**Uncorrected *p*-value**	**% variation**	***n***	**Uncorrected *p*-value**	**% variation**	***n***
**GM density**									
Cerebellum GM	*p* < 0.001 *	−10.7	26	*p* < 0.001 *	−15.4	12	*p* < 0.001 *	−7.7	14
Left putamen	*p* < 0.001 *	−33.1	26	*p* < 0.001 *	−33.0	12	*p* < 0.001 *	−33.2	14
Right putamen	*p* < 0.001 *	−19.8	26	*p* < 0.001 *	−20.9	12	*p* = 0.0052	−18.8	14
**WM density**									
Cerebellum -brainstem	*p* < 0.001 *	−9.3	26	*p* < 0.001 *	−18.0	12	*p* < 0.001 *	−5.0	14
SN	*p* = 0.034	−0.7	26	*p* = 0.0048	−1.7	12	*p* > 0.05	0.1	14
**T1w/T2w**									
Cerebellum GM	*p* = 0.0019 *	6.6	19	*p* = 0.0098	7.5	10	*p* = 0.039	5.7	9
Left putamen	*p* < 0.001 *	24.9	19	*p* = 0.0019 *	28.4	10	*p* = 0.0078	21.3	9
Right putamen	*p* = 0.0037	10.2	19	*p* = 0.0039	12.6	10	*p* > 0.05	7.6	9
Cerebellum-brainstem WM	*p* = 0.012	4.9	19	*p* = 0.049	5.7	10	*p* > 0.05	4.1	9
SN	*p* < 0.001 *	−31.0	19	*p* = 0.0039	−27.6	10	*p* = 0.0039	−35.1	9

Asterisks indicate statistical results significant after Bonferroni correction for multiple comparisons. % variation is calculated as 100*(t1-t0)/t0; n indicates the sample size.

**Figure 2:**
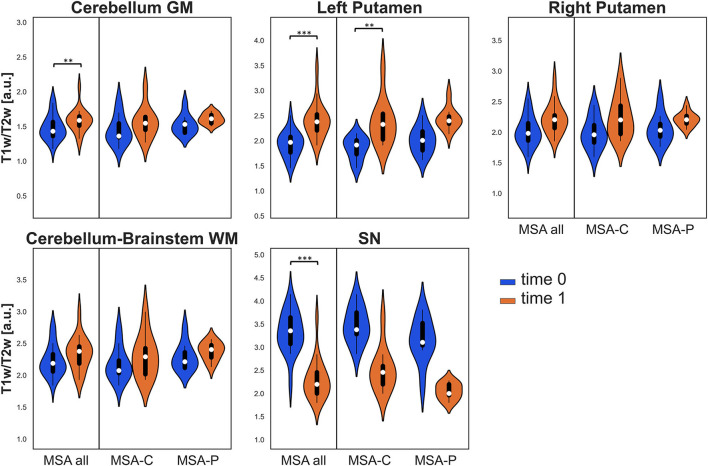
Violin plots describing the distribution of regional T1w/T2w values in all the considered ROIs. Values at baseline (*t*0) are in blue, while values at follow-up (*t*1) are in orange for the entire MSA group and for both MSA subgroups. Significant differences are indicated with asterisks. **p < 0.01, ***p < 0.001.

Due to excessive motion or poor patient compliance, seven patients lacked or had corrupted T2w sequences in at least one of the two evaluations; therefore, the same clusters assessed for VBM were also studied using the T1w/T2w ratio only in 19 patients with MSA (10 MSA-C and 9 MSA-P). In this case, when considering the entire MSA group, a significant longitudinal increase was detected in the clusters of cerebellar GM and left putamen (where a significant increase in T1w/T2w ratio was also found in the MSA-C subgroup), while a significant decrease was detected in the bilateral SN (refer to Table 2 and Figure 2).

MRI parameter longitudinal variation was correlated with a clinical longitudinal variation using Spearman's rank correlation coefficient. The variation in WM density (between time points) in the cluster of cerebellum-brainstem was found to be negatively correlated to variations in UMSARS-I score when considering the entire MSA group (*p* = 0.014, ρ = −0.48) and for both MSA subgroups separately (MSA-P: *p* = 0.044, ρ = −0.55; MSA-C: *p* = 0.045, ρ = −0.59). In both the entire MSA group and the MSA-P subgroup, the longitudinal variation of WM density in bilateral SN was found to negatively correlate with variations in UMSARS-I score (MSA: *p* = 0.0017, ρ = −0.58; MSA-P: *p* < 0.001, ρ = −0.78) and in UMSARS-II (MSA: *p* = 0.0088, ρ = −0.50; MSA-P: *p* = 0.049, ρ = −0.53), refer to Supplementary Figure S2 for the scatterplots of the significant correlations. None of the other explored correlations were found significant, and the complete results can be found in Supplementary Table S1.

Finally, a general linear model was performed to assess how the MRI parameters (GM, WM density, and T1w/T2w) at baseline influence the clinical changes (in terms of UMSARS scores variation), accounting for disease duration. The general linear regression model showed that in the MSA-P subgroup, the GM density at t0 in left and right putamina predicted the score variation over time as expressed by ΔUMSARS-II (left putamen: *p* = 0.0057, *t* = −3.42; right putamen: *p* = 0.021, *t* = −2.69) and ΔUMSARS-IV (left putamen: *p* = 0.0092, *t* = −3.15; right putamen: *p* = 0.0020, *t* = −4.02). Moreover, in the MSA-P subgroup, the T1w/T2w value at t0 in the left putamen predicted the ΔUMSARS-IV score variation over time (*p* = 0.032, *t* = 2.78). All the weights from the general linear regression models can be found in Supplementary Table S2.

## Discussion

In this study, we analyzed regional brain atrophy and T1w/T2w ratio in order to characterize the longitudinal brain changes in a group of patients with MSA in a 1-year follow-up. Significant changes were detected both in GM and WM atrophy and in T1w/T2w values, and some of these changes were associated with the worsening of functional disability and motor symptoms as expressed by UMSARS-I and UMSARS-II scores.

In particular, a significant progression of atrophy was found in cerebellar and putaminal GM and in cerebellum/brainstem WM in both MSA-P and MSA-C at follow-up. In previous 1-year follow-up VBM studies assessing smaller cohorts of patients and not analyzing MSA subtypes separately, significant progression of atrophy was found in the striatum and cerebellum of patients with MSA and not in age-matched patients with PD or HCs (4, 8). In our study, the percent variability of atrophy (over the follow-up period) ranged from approximately 9% in the cerebellum-brainstem WM to 33% in the left putamen in the entire MSA group. The higher annual rate of atrophy in cerebellar GM in MSA-C as compared with MSA-P suggests that more progressive atrophy of the cerebellum is a feature of the MSA-C subtype, regardless of a similar increase of putaminal atrophy in both subtypes. In a recent study evaluating GM and WM abnormalities in early MSA-C and MSA-P (mean disease duration 1.6 years) by means of VBM and diffusion tensor imaging, in the MSA-C subtype, GM loss was limited to the cerebellum with WM changes mostly affecting the infratentorial WM, while in MSA-P, no GM loss was found, and WM involvement was mainly supratentorial (23). In contrast, an automated region-based volumetric analysis comparing the progression of subcortical atrophy in 8 patients with MSA-P and 9 patients with MSA-C showed more rapid progression of putamen atrophy in patients with MSA-P than in patients with MSA-C. In fact, in this study, patients had a shorter disease duration (approximately 2 years) and a longer scan interval (18 months) than our study, and a different measure of subcortical atrophy was used (volume), while cerebellum atrophy was not assessed at all (24).

When assessing T1w/T2w changes as a potential MRI marker of disease progression, we found that this ratio significantly increased in the cerebellar GM and left putamen in the entire MSA group, possibly corresponding to the decrease in neuronal density or iron accumulation, that is known to occur in the putamen of patients with MSA (25). In particular, the percent variation over time was higher in left putamen (25%) than in cerebellar GM (approximately 7%). In contrast, the T1w/T2w ratio showed a significant decrease at follow-up in the bilateral SN, a finding that could be explained by a loss of myelin integrity over time or a progressive change in dendrite density as shown in the cerebral cortex in a pathological study on multiple sclerosis (26). Interestingly, VBM did not show a significant progression of SN atrophy over time, and therefore, the T1w/T2w ratio, with a 30% variation at 1-year follow-up, could be an MRI parameter more sensitive to change in this brain area. The rationale for studying SN resides in its involvement in MSA as known by neuropathological studies (27, 28). In fact, regional neurodegeneration in SN as assessed by neuromelanin-sensitive MRI does not differ between MSA and PD, and the T1w/T2w ratio in SN has been recently proposed as a novel parsimonious biomarker in the PD population (15).

The T1w/T2w ratio can be considered an easy-to-implement clinically feasible measure of tissue microstructural integrity, already used in several neurological disorders and possibly reflecting demyelination, inflammation, gliosis, as well as axonal and dendritic injury (10, 15, 29). Even though our results need further validation in independent studies, these data suggest that T1w/T2w may be a useful MRI marker of disease progression in MSA.

We found that a greater reduction of WM density in the cerebellum-brainstem was associated with a greater worsening of functional scores, as assessed by UMSARS I, in both MSA subtypes. Moreover, a greater longitudinal reduction of WM density in bilateral SN was related to a greater worsening of both functional and motor scores (as assessed by UMSARS II) in the entire MSA group and in MSA-P.

This important aspect suggests that pathological involvement of the WM structures of cerebellum-brainstem and SN is associated with worsening in the clinical features of the disease and supports the further study of such MRI parameters as surrogate biomarkers of MSA progression. In fact, both WM abnormalities and nigral alterations are interesting markers that are being studied in MSA by means of different techniques with the main purpose of differential diagnosis (30, 31).

In contrast, we found that higher putaminal GM density and T1w/T2w ratio at baseline, respectively, predicted less and higher deterioration of functional, motor, and global disability scores at follow-up only in MSA-P, suggesting that mainly the changes in putaminal integrity are associated with clinical progression in MSA-P, consistently with pathological data (32).

We recognized that the fact that WM changes in cerebellum/brainstem and SN, but not GM/putamen changes correlated with clinical variable changes in the entire MSA population may be unexpected. However, we know that MSA is a unique proteinopathy that differs from other α-synucleinopathies since the pathological process resulting from the accumulation of aberrant α-synuclein (αSyn) primarily involves the oligodendroglia rather than neurons (33), and WM changes in MSA are being increasingly recognized in imaging studies (34–36). Further studies are needed to understand if WM changes in the cerebellum/brainstem and SN are more related to clinical variations in MSA than the more commonly studied GM putaminal density.

Our study presents some important limitations. First, the sample size is relatively small, especially when considering clinical subtypes separately, and second, we lack an HC group with longitudinal scans. However, difficulties in patient recruiting and realizing a longitudinal study due to disease rarity and rapidly progressive course should also be acknowledged. Future studies are needed to validate our results on larger samples (possibly through multicenter studies), also including HCs for longitudinal examination. Moreover, we recognized that, possibly due to the limited sample size, some longitudinal variations, although statistically significant, remain small in terms of effect size (in this study, expressed as percent variation of the parameters between scans), and thus, not all the considered ROIs can represent robust biomarkers of disease progression.

We also need to acknowledge the controversies on T1w/T2w maps as a marker of brain myelin content (37, 38), thus additional investigations in combination with postmortem histological analysis should be performed in order to possibly dig into the relationship between this MRI parameter and the underlying tissue characteristics and disease-related changes.

To validate new therapies in the course of clinical trials, we need morphological markers that reflect disease progression. Our results shed light on specific MRI markers that are potentially sensitive to change over time in MSA. In particular, the T1w/T2w ratio is a measure that could be used as an objective marker to monitor disease progression after a short follow-up period, with putamen and SN showing the most relevant changes at 1-year follow-up. As for VBM, it usually requires group comparisons; however, an individual adjusting covariate method has been just recently proposed to enable subject-specific atrophy evaluation and eventually diagnosis from VBM data (39). Further validation is needed to establish the value of these MRI measures as progression biomarkers in MSA and their potential implication for future therapeutic trials.

## Data availability statement

The raw data supporting the conclusions of this article will be made available by the authors, without undue reservation.

## Ethics statement

The studies involving human participants were reviewed and approved by Comitato Etico Campania Sud. The patients/participants provided their written informed consent to participate in this study.

## Author contributions

SP performed MRI data processing, analysis, and wrote the first draft of the manuscript. RM, GD, FD, and FE critically revised MRI analyses and the final version of the manuscript. MR, VA, LF, RE, MA, SC, MP, and PB collected clinical data and critically revised the final version of the manuscript. MP collected clinical data and wrote the final version of the manuscript. All authors contributed to the article and approved the submitted version.

## Conflict of interest

The authors declare that the research was conducted in the absence of any commercial or financial relationships that could be construed as a potential conflict of interest.

## Publisher's note

All claims expressed in this article are solely those of the authors and do not necessarily represent those of their affiliated organizations, or those of the publisher, the editors and the reviewers. Any product that may be evaluated in this article, or claim that may be made by its manufacturer, is not guaranteed or endorsed by the publisher.
