# Supplementary material for: Combined regional T1w/T2w ratio and voxel-based morphometry in multiple system atrophy: A follow-up study
Source: Front Neurol. 2022 Oct 19;13:1017311. doi: 10.3389/fneur.2022.1017311 (PMC9626981; doi:10.3389/fneur.2022.1017311)
Supplement: Supplementary file 1 [file Table_1.DOCX]

Supplementary Table 1. Results of correlation analysis

|  | **UMSARS I** | | **UMSARS II** | | **UMSARS IV** | |
| --- | --- | --- | --- | --- | --- | --- |
|  | ρ | p-value | ρ | p-value | ρ | p-value |
|  | **MSA entire group** | | | | | |
| **GM density** |  |  |  |  |  |  |
| Cerebellum GM | -0.11 | p>0.05 | -0.09 | p>0.05 | 0.04 | p>0.05 |
| Left Putamen | 0.15 | p>0.05 | 0.26 | p>0.05 | 0.15 | p>0.05 |
| Right Putamen | 0.29 | p>0.05 | 0.29 | p>0.05 | 0.17 | p>0.05 |
| **WM density** |  |  |  |  |  |  |
| Cerebellum-Brainstem | **-0.48** | **p=0.014** | -0.30 | p>0.05 | -0.35 | p>0.05 |
| SN | **-0.58** | **p=0.0017** | **-0.50** | **p=0.0088** | -0.26 | p>0.05 |
| **T1w/T2w** |  |  |  |  |  |  |
| Cerebellum GM | -0.23 | p>0.05 | -0.22 | p>0.05 | -0.07 | p>0.05 |
| Left Putamen | 0.35 | p>0.05 | 0.05 | p>0.05 | 0.26 | p>0.05 |
| Right Putamen | 0.13 | p>0.05 | -0.11 | p>0.05 | 0.03 | p>0.05 |
| Cerebellum-Brainstem WM | -0.07 | p>0.05 | -0.13 | p>0.05 | -0.07 | p>0.05 |
| SN | 0.29 | p>0.05 | 0.17 | p>0.05 | 0.06 | p>0.05 |
|  | **MSA-C** | | | | | |
| **GM density** |  |  |  |  |  |  |
| Cerebellum GM | -0.02 | p>0.05 | 0.11 | p>0.05 | 0.21 | p>0.05 |
| Left Putamen | -0.09 | p>0.05 | -0.08 | p>0.05 | -0.08 | p>0.05 |
| Right Putamen | 0.20 | p>0.05 | 0.18 | p>0.05 | 0.15 | p>0.05 |
| **WM density** |  |  |  |  |  |  |
| Cerebellum -Brainstem | **-0.59** | **p=0.045** | -0.21 | p>0.05 | -0.47 | p>0.05 |
| SN | -0.35 | p>0.05 | -0.48 | p>0.05 | -0.29 | p>0.05 |
| **T1w/T2w** |  |  |  |  |  |  |
| Cerebellum GM | -0.40 | p>0.05 | -0.34 | p>0.05 | -0.12 | p>0.05 |
| Left Putamen | 0.45 | p>0.05 | 0.06 | p>0.05 | 0.52 | p>0.05 |
| Right Putamen | 0.37 | p>0.05 | -0.01 | p>0.05 | 0.46 | p>0.05 |
| Cerebellum-Brainstem WM | 0.10 | p>0.05 | -0.06 | p>0.05 | -0.22 | p>0.05 |
| SN | 0.08 | p>0.05 | -0.21 | p>0.05 | -0.38 | p>0.05 |
|  | **MSA-P** | | | | | |
| **GM density** |  |  |  |  |  |  |
| Cerebellum GM | -0.21 | p>0.05 | -0.26 | p>0.05 | 0.1949 | p>0.05 |
| Left Putamen | 0.33 | p>0.05 | 0.45 | p>0.05 | 0.5139 | p>0.05 |
| Right Putamen | 0.44 | p>0.05 | 0.51 | p>0.05 | 0.4075 | p>0.05 |
| **WM density** |  |  |  |  |  |  |
| Cerebellum-Brainstem | **-0.55** | **p=0.044** | -0.50 | p>0.05 | -0.159 | p>0.05 |
| SN | **-0.78** | **p<0.001** | **-0.53** | **p=0.049** | -0.23 | p>0.05 |
| **T1w/T2w** |  |  |  |  |  |  |
| Cerebellum GM | -0.04 | p>0.05 | 0.09 | p>0.05 | 0 | p>0.05 |
| Left Putamen | 0.25 | p>0.05 | 0.24 | p>0.05 | 0 | p>0.05 |
| Right Putamen | 0.00 | p>0.05 | -0.01 | p>0.05 | -0.433 | p>0.05 |
| Cerebellum-Brainstem WM | -0.23 | p>0.05 | -0.05 | p>0.05 | 0.1732 | p>0.05 |
| SN | 0.48 | p>0.05 | 0.56 | p>0.05 | 0.52 | p>0.05 |

Results of the correlation analysis between longitudinal variation of regional values of GM, WM density and T1w/T2w ratio and changes in UMSARS scores. Spearman’s correlations were calculated considering the entire MSA group as well as separating subgroups (MSA-P and MSA-C). Significant (uncorrected) correlations were indicated in bold.

Supplementary Table 2. Results of the linear regression model.

| 1. **Tissue density values (GM and WM)** | | | |
| --- | --- | --- | --- |
| **GLM formula – MSA-all** | **α** | **β** | **γ** |
| ΔUMSARS-I = α Cerebellum GM + β disease duration + γ | 0.66 | -043 | 6.48 |
| ΔUMSARS-I = α LPutamen + β disease duration + γ | -6.87 | -0.34 | 9.96 |
| ΔUMSARS-I = α RPutamen + β disease duration + γ | -8.89 | -0.40 | 11.03 |
| ΔUMSARS-I = α CerebellumBrainstem WM + β disease duration + γ | -0.95 | -0.44 | 7.12 |
| ΔUMSARS-I = α SN + β disease duration + γ | -5.32 | -0.47 | 11.50 |
| ΔUMSARS-II = α Cerebellum GM + β disease duration + γ | -9.02 | -0.84 | 11.93 |
| ΔUMSARS-II = α LPutamen + β disease duration + γ | -12.97 | -0.66 | 13.94 |
| ΔUMSARS-II = α RPutamen + β disease duration + γ | -13.56 | -0.78 | 14.40 |
| ΔUMSARS-II = α CerebellumBrainstem WM + β disease duration + γ | -8.23 | -0.88 | 10.94 |
| ΔUMSARS-II = α SN + β disease duration + γ | -15.42 | -0.93 | 21.60 |
| ΔUMSARS-IV = α Cerebellum GM + β disease duration + γ | -1.47 | -0.096 | 1.64 |
| ΔUMSARS-IV = α LPutamen + β disease duration + γ | -0.56 | -0.087 | 1.25 |
| ΔUMSARS-IV = α RPutamen + β disease duration + γ | -0.92 | -0.091 | 1.43 |
| ΔUMSARS-IV = α CerebellumBrainstem WM + β disease duration + γ | -1.12 | -0.10 | 1.40 |
| ΔUMSARS-IV = α SN + β disease duration + γ | -2.60 | -0.11 | 3.30 |
| **GLM formula – MSA-C** |  |  |  |
| ΔUMSARS-I = α Cerebellum GM + β disease duration + γ | 26.36 | -1.13 | 0.46 |
| ΔUMSARS-I = α LPutamen + β disease duration + γ | 16.59 | -1.43 | 1.46 |
| ΔUMSARS-I = α RPutamen + β disease duration + γ | 14.62 | -1.21 | 2.06 |
| ΔUMSARS-I = α CerebellumBrainstem WM + β disease duration + γ | -7.29 | -1.03 | 11 |
| ΔUMSARS-I = α SN + β disease duration + γ | 4.09 | -1.09 | 6.02 |
| ΔUMSARS-II = α Cerebellum GM + β disease duration + γ | 8.60 | -1.40 | 7.83 |
| ΔUMSARS-II = α LPutamen + β disease duration + γ | 6.08 | -1.45 | 6.08 |
| ΔUMSARS-II = α RPutamen + β disease duration + γ | -0.98 | -1.31 | 11.23 |
| ΔUMSARS-II = α CerebellumBrainstem WM + β disease duration + γ | -8.20 | -1.26 | 12.55 |
| ΔUMSARS-II = α SN + β disease duration + γ | -2.08 | -1.32 | 12.45 |
| ΔUMSARS-IV = α Cerebellum GM + β disease duration + γ | -2.20 | -0.15 | 1.44 |
| ΔUMSARS-IV = α LPutamen + β disease duration + γ | 2.52 | -0.20 | 0.17 |
| ΔUMSARS-IV = α RPutamen + β disease duration + γ | 2.87 | -0.17 | 0.06 |
| ΔUMSARS-IV = α CerebellumBrainstem WM + β disease duration + γ | -2.53 | -0.13 | 1.93 |
| ΔUMSARS-IV = α SN + β disease duration + γ | -1.59 | -0.15 | 2.68 |
| **GLM formula – MSA-P** |  |  |  |
| ΔUMSARS-I = α Cerebellum GM + β disease duration + γ | 0.60 | 0.14 | 4.18 |
| ΔUMSARS-I = α LPutamen + β disease duration + γ | -17.77 | 0.24 | 12.55 |
| ΔUMSARS-I = α RPutamen + β disease duration + γ | -19.80 | 0.11 | 13.59 |
| ΔUMSARS-I = α CerebellumBrainstem WM + β disease duration + γ | 9.26 | 0.32 | -0.04 |
| ΔUMSARS-I = α SN + β disease duration + γ | -8.65 | 0.020 | 12.65 |
| ΔUMSARS-II = α Cerebellum GM + β disease duration + γ | -5.85 | -0.46 | 8.62 |
| ΔUMSARS-II = α LPutamen + β disease duration + γ | **-27.21** | -0.25 | 17.84 |
| ΔUMSARS-II = α RPutamen + β disease duration + γ | **-26.92** | -0.44 | 17.86 |
| ΔUMSARS-II = α CerebellumBrainstem WM + β disease duration + γ | -1.79 | -0.43 | 6.38 |
| ΔUMSARS-II = α SN + β disease duration + γ | -17.53 | -0.64 | 22 |
| ΔUMSARS-IV = α Cerebellum GM + β disease duration + γ | -0.43 | -0.05 | 0.90 |
| ΔUMSARS-IV = α LPutamen + β disease duration + γ | **-2.64** | -0.032 | 1.86 |
| ΔUMSARS-IV = α RPutamen + β disease duration + γ | **-3.42** | -0.052 | 2.24 |
| ΔUMSARS-IV = α CerebellumBrainstem WM + β disease duration + γ | 1.32 | -0.021 | 0.02 |
| ΔUMSARS-IV = α SN + β disease duration + γ | -2.04 | -0.075 | 2.59 |
| 1. **T1w/T2w values** |  |  |  |
| **GLM formula – MSA-all** | **α** | **β** | **γ** |
| ΔUMSARS-I = α Cerebellum GM + β disease duration + γ | 0.028 | -0.13 | 5.76 |
| ΔUMSARS-I = α LPutamen + β disease duration + γ | 2.60 | -0.058 | 0.51 |
| ΔUMSARS-I = α RPutamen + β disease duration + γ | 6.56 | 0.014 | -7.97 |
| ΔUMSARS-I = α CerebellumBrainstem WM + β disease duration + γ | -0.072 | -0.13 | 5.97 |
| ΔUMSARS-I = α SN + β disease duration + γ | -2.10 | -0.0051 | 12.33 |
| ΔUMSARS-II = α Cerebellum GM + β disease duration + γ | -0.54 | -0.69 | 8.58 |
| ΔUMSARS-II = α LPutamen + β disease duration + γ | 7.33 | -0.48 | -7.15 |
| ΔUMSARS-II = α RPutamen + β disease duration + γ | 7.24 | -0.52 | -7.48 |
| ΔUMSARS-II = α CerebellumBrainstem WM + β disease duration + γ | -1.56 | -0.73 | 11.40 |
| ΔUMSARS-II = α SN + β disease duration + γ | 0.51 | -0.71 | 6.16 |
| ΔUMSARS-IV = α Cerebellum GM + β disease duration + γ | -0.062 | 0.071 | 0.45 |
| ΔUMSARS-IV = α LPutamen + β disease duration + γ | 0.069 | 0.074 | 0.22 |
| ΔUMSARS-IV = α RPutamen + β disease duration + γ | 0.30 | 0.079 | -0.27 |
| ΔUMSARS-IV = α CerebellumBrainstem WM + β disease duration + γ | -0.011 | 0.072 | 0.38 |
| ΔUMSARS-IV = α SN + β disease duration + γ | 0.011 | 0.071 | 0.32 |
| **GLM formula – MSA-C** |  |  |  |
| ΔUMSARS-I = α Cerebellum GM + β disease duration + γ | 2.60 | -0.71 | 4.24 |
| ΔUMSARS-I = α LPutamen + β disease duration + γ | 2.93 | -0.79 | 2.89 |
| ΔUMSARS-I = α RPutamen + β disease duration + γ | 3.48 | -0.68 | 0.98 |
| ΔUMSARS-I = α CerebellumBrainstem WM + β disease duration + γ | 2.02 | -0.70 | 3.55 |
| ΔUMSARS-I = α SN + β disease duration + γ | 5.83 | -1.65 | -8.71 |
| ΔUMSARS-II = α Cerebellum GM + β disease duration + γ | 5.13 | -0.61 | 0.37 |
| ΔUMSARS-II = α LPutamen + β disease duration + γ | 6.56 | -0.78 | -3.66 |
| ΔUMSARS-II = α RPutamen + β disease duration + γ | 6.12 | -0.56 | -4.53 |
| ΔUMSARS-II = α CerebellumBrainstem WM + β disease duration + γ | 3.28 | -0.59 | 0.58 |
| ΔUMSARS-II = α SN + β disease duration + γ | 9.31 | -2.11 | -18.86 |
| ΔUMSARS-IV = α Cerebellum GM + β disease duration + γ | 0.19 | -0.037 | 0.56 |
| ΔUMSARS-IV = α LPutamen + β disease duration + γ | -0.069 | -0.037 | 0.97 |
| ΔUMSARS-IV = α RPutamen + β disease duration + γ | 0.15 | -0.036 | 0.54 |
| ΔUMSARS-IV = α CerebellumBrainstem WM + β disease duration + γ | 0.27 | -0.034 | 0.23 |
| ΔUMSARS-IV = α SN + β disease duration + γ | 0.98 | -0.19 | -1.98 |
| **GLM formula – MSA-P** |  |  |  |
| ΔUMSARS-I = α Cerebellum GM + β disease duration + γ | -3.77 | 1.35 | 5.96 |
| ΔUMSARS-I = α LPutamen + β disease duration + γ | 7.71 | 2.80 | -20.78 |
| ΔUMSARS-I = α RPutamen + β disease duration + γ | 11.55 | 2.074 | -26.21 |
| ΔUMSARS-I = α CerebellumBrainstem WM + β disease duration + γ | -2.46 | 1.39 | 5.69 |
| ΔUMSARS-I = α SN + β disease duration + γ | -6.39 | 0.12 | 25.03 |
| ΔUMSARS-II = α Cerebellum GM + β disease duration + γ | -14.07 | -1.56 | 32.13 |
| ΔUMSARS-II = α LPutamen + β disease duration + γ | 13.63 | 1.39 | -27.92 |
| ΔUMSARS-II = α RPutamen + β disease duration + γ | 9.57 | 9.57 | -13.45 |
| ΔUMSARS-II = α CerebellumBrainstem WM + β disease duration + γ | -9.58 | -1.45 | 32.15 |
| ΔUMSARS-II = α SN + β disease duration + γ | -5.83 | -2.11 | 31.15 |
| ΔUMSARS-IV = α Cerebellum GM + β disease duration + γ | 0.15 | 0.39 | -1.15 |
| ΔUMSARS-IV = α LPutamen + β disease duration + γ | **1.23** | 0.58 | -4.10 |
| ΔUMSARS-IV = α RPutamen + β disease duration + γ | 0.73 | 0.41 | -2.51 |
| ΔUMSARS-IV = α CerebellumBrainstem WM + β disease duration + γ | -0.055 | 0.38 | -0.74 |
| ΔUMSARS-IV = α SN + β disease duration + γ | -0.32 | 0.31 | 0.40 |

Results of the general linear models to assess how the MRI parameters at baseline influences the clinical changes (in terms of ΔUMSARS scores variation), accounting for disease duration; a) GM, WM density in and b) T1w/T2w values. For each model, the estimate of the three weights are presented in the columns α, β and γ, respectively the weight for the MRI parameter, the disease duration and the intercept.

Supplementary Figure 1.


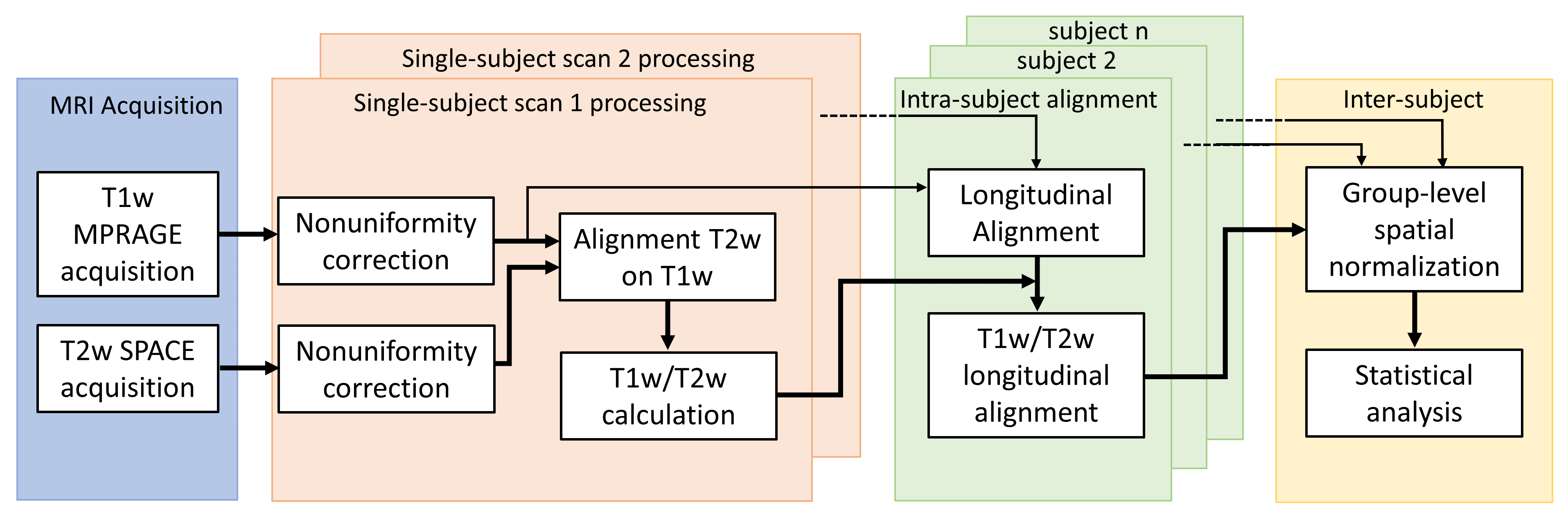


Supplementary Figure1. Workflow of T1w/T2w image analysis from acquisition to statistical analysis. After image acquisition (blue box), each image in corrected for nonuniform intensity, the map is calculated for each subject and each scan (orange box); then the intra-subject longitudinal alignment is performed for each subject (green box) and finally the group-level spatial normalization is carried out to perform group analysis and statistical comparison (yellow box).

Supplementary Figure 2


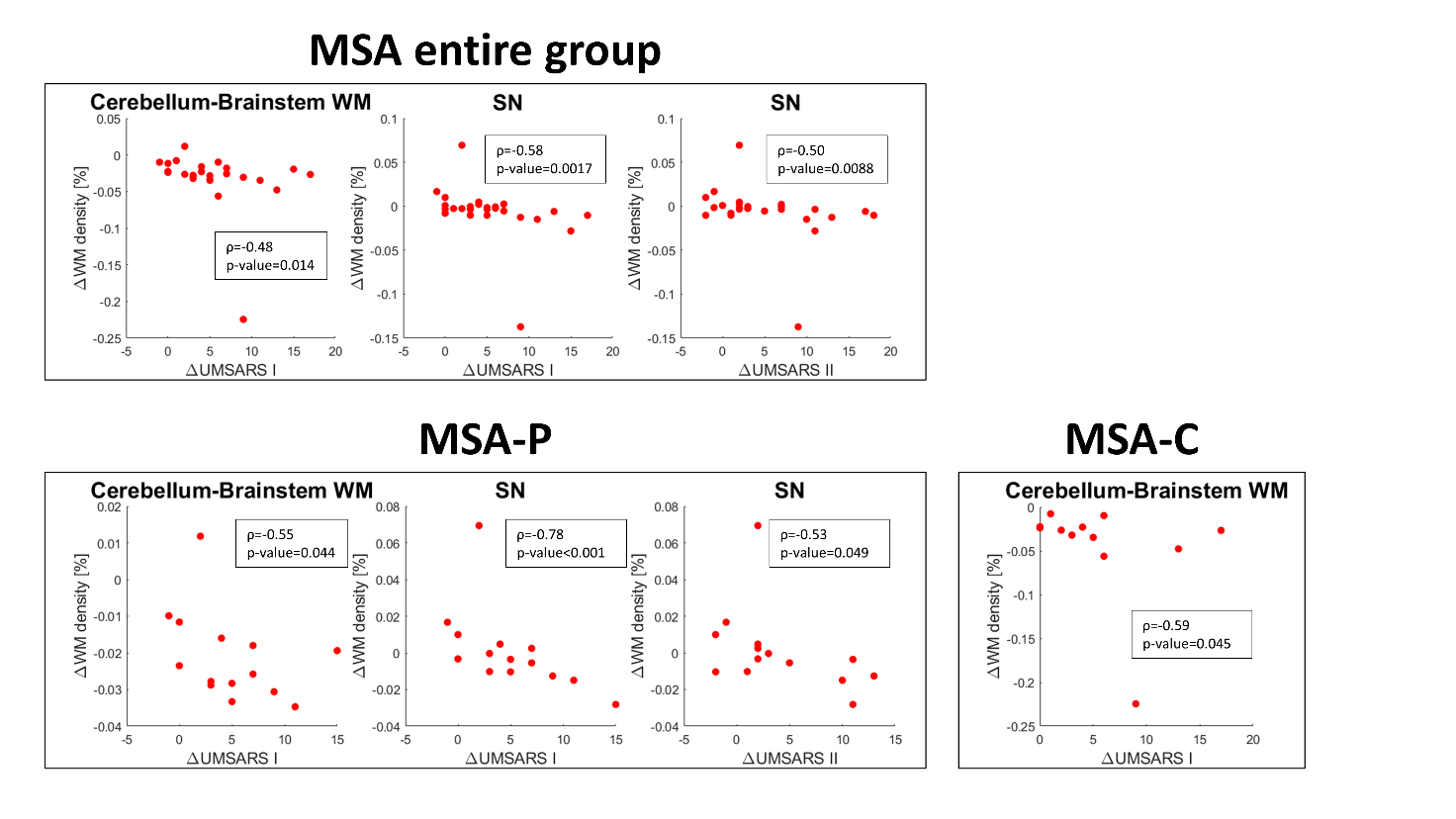


Supplementary Figure 2. Scatter plot and results of Spearman correlation (ρ and p-value) of the MRI parameters vs clinical scores (UMSARS scale) in the ROIs of significant correlation.
